# HPV immunization in Brazil and proposals to increase adherence to vaccination campaigns

**DOI:** 10.11606/s1518-8787.2023057005410

**Published:** 2023-10-24

**Authors:** Wagner Mesojedovas Santos, Debora Mesojedovas Santos, Márcia Santana Fernandes

**Affiliations:** I Universidade Federal do Rio Grande do Sul Hospital de Clínicas de Porto Alegre Programa de Pós-graduação em Pesquisa Clínica Porto Alegre RS Brasil Universidade Federal do Rio Grande do Sul. Hospital de Clínicas de Porto Alegre. Programa de Pós-graduação em Pesquisa Clínica. Porto Alegre, RS, Brasil; II Universidade Federal do Rio Grande do Sul Hospital de Clínicas de Porto Alegre Laboratório de Pesquisa de Bioética e Ética na Ciência Porto Alegre RS Brasil Universidade Federal do Rio Grande do Sul. Hospital de Clínicas de Porto Alegre. Laboratório de Pesquisa de Bioética e Ética na Ciência. Porto Alegre, RS, Brasil

**Keywords:** Human papillomavirus, Uterine Cervical Neoplasms, Social Marketing, Immunization, Immunization Programs

## Abstract

**OBJECTIVES:**

To identify the possible causes of low adherence to vaccination campaigns in Brazil, find and analyze campaigns regarding human papillomavirus (HPV) in Brazil and abroad, and apply quality tools to prepare proposals to increase vaccination coverage (VC) and prevent HPV in the country.

**METHOD:**

This is a qualitative and deductive-hypothetical research. A narrative review of the literature (especially on the narratives and formats applied in vaccination campaigns in Brazil) was the technique used to develop our method.

**RESULTS:**

Brazil had a 49.6% VC in 2019, unlike countries such as Australia (80.2% in 2017), Mexico (97.5% in 2019), and Peru (91% in 2019). This study found evidence of the use of social marketing strategies to engage communities as good practices in the vaccination campaigns of these countries.

**CONCLUSION:**

With the retrieved information, three quality tools (Ishikawa diagram, Pareto graph, and 5W2H) classified and quantified the causes of low VC in Brazil and enabled proposals that can direct its Ministry of Health to take more effective strategies to achieve the HPV VC goal recommended by the WHO.

## INTRODUCTION

Sexual contact constitutes the main transmission route for human papillomavirus (HPV). Among the more than 100 types of HPV in the medical literature, 50 affect the mucosa of the genital tract and at least 15 are classified as high risk, i.e., with carcinogenic potential ^1^ . High-risk HPV causes 99% of cervical cancer cases, with type 16 being the most commonly variation detected in carcinoma ^1^ and among the four types of cancers that kill the most per year in Brazil (6,526 deaths in 2019) ^2^ . The world endured 311,000 in 2018 ^3^ . Among all types of cancer, cervical cancer is the only one that currently has an immunizer as preventive.

HPV types 16 and 18 account for about 70% of cervical cancer cases; whereas types 6 and 11, for more than 90% of all genital warts ^4^ . These four types are prophylactic targets of the recombinant quadrivalent vaccine to prevent HPV — distributed by the National Immunization Program ( *Programa Nacional de Imunização* – PNI) of the Brazilian Unified Health System ( *Sistema Único de Saúde* – SUS) since 2014. Brazil has a vaccination coverage (VC) target of 80%, aligned with the World Health Organization (WHO). However, seven years after the beginning of the national vaccination campaign to prevent HPV (2014–2021), Ministry of Health (MH) data show that the country has failed to reach its goal ^5^ .

The health systems of several countries have adopted vaccination campaigns to prevent HPV following WHO goals and recommendations. Currently, 114 nations have included two or three doses in the vaccination schedule of their public health programs ^3^ . Although the WHO recommends 80% vaccination coverage for target audiences in all countries, some have VCs below the recommended target, such as Ireland (73%) and Switzerland (58.9%). On the other hand, countries such as Australia, Mexico, and Peru have VCs above the WHO target ^6^ . In general, different communication channels and the literature show that these countries have used social marketing techniques in their vaccination campaigns to better engage their target audience.

Social marketing consists of a set of initiatives with a social purpose whose main objective is to modify behavior and influence social issues by several approaches without focusing on the trade of goods or services ^7^ . Kotler (creator of social marketing) highlights the impossibility of influencing people’s behavior without considering the socioeconomic aspect. Social marketing also argues for the fundamental importance of the ability to generate inputs and tools to develop effective interventions, stressing that the focus lies on individuals, groups, or society’s well-being. Social marketing can be applied based on Kotler’s four “P” (product, price, place, and promotion). The four “P” provide a sort of guide to the success of a social marketing strategy, which Nowak considers the heart of social marketing ^8^ . Therefore, it is inferred that countries with vaccination targets far below those recommended by the WHO (e.g., Brazil) fail to use the benefits of social marketing, especially the four benefits that have direct link with the four “P”: offer of real benefits, costs, ease of access, and personalized messages.

Nevertheless, the social marketing from the vaccination campaigns of Australia, Mexico, and Peru can be considered as an opportunity to use quality tools to identify problems, causes, and possible solutions to improve VC in Brazil. Quality tools constitute instruments that aim to facilitate the various activities of a corporation to better manage its processes ^9^ .

The Ishikawa diagram is such an instrument, a process management quality tool that requires the mapping of the delivery of products or services within defined parameters. Also known as cause and effect or fishbone diagram, the Ishikawa diagram is a visual tool that helps with problem analysis ^9^ and enables the quick identification of the root cause of problems to mitigate them.

Another quality tool is the Pareto chart, which allows us to prioritize and address problems to quickly obtain significant results without the need to mitigate all causes at once. The Pareto chart is known as the 80-20 chart as it is based on the rule that 80% of results stem from 20% of actions taken. It enables to focus actions on 20% of the main problems to achieve 80% of results ^9^ .

Finally, 5W2H is a simple technique that can guide people in situations and problems by generating an action plan for each problem. The tool has seven questions, creating a roadmap that can evaluate problems and their impact to offer solutions. The questions are *what, why, where, when, who, how,* and *how much it costs* ^9^ .

This literature review aimed to 1) identify the possible causes for Brazil failing to reach its goal of 80% immunization and 2) to list the main strategies and tactics of vaccination adherence campaigns toward the prevention of HPV in other countries to serve as good adherence practices in Brazil based on the social marketing approach. Finally, this study aimed to 3) apply quality tools to classify and quantify the causes of low VC and enable proposals that can guide the Brazilian Ministry of Health to take more effective strategies to achieve the VC goal recommended by the WHO.

## METHODS

1) To identify the causes of insufﬁcient VC in Brazil, a bibliographic and documentary research was adopted to collect information for our analyses on vaccination campaigns in Brazil and abroad using public domain websites. A narrative review of the medical literature was also carried out using keywords such as “human papillomavirus,” “vaccination,” and “HPV vaccination campaign” in databases such as PubMed, Embase, Scopus, Bireme, and SciELO (Jan./2010–Dec./2022).

To collect information and data on the Brazilian vaccination campaign (2014–2021), official data available on the “access to information” tab on the MH website (https://falabr.cgu.gov.br/) were used. Public data available on the PNI portal (http://pni.datasus.gov.br/) were also included, as were complementary information on 2019, which was accessed via the Brazilian Society of Clinical Oncology ( *Sociedade Brasileira de Oncologia Clínica* – SBOC) website. The data were separated according to coverage for the first and second doses of the vaccine and a simple mean for all genders was calculated to assess overall coverage and evaluate it according to the goal recommended by the MH.

2) To find the main strategies and tactics of vaccination adherence campaigns carried out globally according to social marketing, it was necessary to collect VC data from other countries, obtained from the official report on indicators from the WHO (The Global Health Observatory) ^3^ . These data were evaluated following published studies. Moreover, three countries—whose information on vaccination campaigns were disseminated and which managed to maintain the goal recommended by the WHO on effective immunization to eradicate cancer—were chosen for comparison. Information on VC and related campaigns was evaluated to find the possible problems in the Brazilian HPV vaccination campaign. The social marketing used by the MH was evaluated according to the theory advocated and defended by Philip Kotler ^7^ and the strategies used in published studies.

Finally, 3) content analysis of all the organized material was performed to translate it into interpretative results using the Ishikawa diagram, Pareto chart, and 5W2H ^9^ to better describe the information.

## RESULTS

### HPV Vaccine Coverage in Brazil

A 49.6% VC in Brazil was determined based on MH, PNI, and SBOC data ( Table ). MH data consider the period from 2014 to 2020 in absolute values without stratifying annual coverages. For 2014 and 2015, the MH vaccination schedule totaled three doses in an interval of zero, two, and six months. From 2016 onward, the regimen changed administration to two doses with a minimum interval of six months. The target audience set for the start of the program in 2014 consisted of girls aged from 11 to 14 years, which changed in 2015 as the program included boys and set its lowest limit to nine years of age, which remains to this day.

**Table:** Vaccination coverage with the first and second doses (D1 and D2) of the quadrivalent HPV vaccine in boys and girls aged from 9 to 14 years in Brazil from 2014 to 2020.

Gender	Target population	D1(%)	D2 (%)	Mean (%)
Girls	9–14 years	82.1	54.5	68.3
Boys	9–14 years	38.6	23.5	31
Total average		60.3	39	49.6

Source: SIPNI TABNET BD – MS (January/21). HPV: human papillomavirus; D1: first dose; D2: second dose.

To calculate VC in girls, the considered period spanned from March 2014 to November 2020; whereas, for boys, from March 2015 to November 2020.

### Reasons for HPV Vaccine Hesitancy and Refusal

Our bibliographical, documental, and medical literature review chose two real-life publications that applied questionnaires to guardians when they refused to vaccinate their children and adolescents.

In September 2010, Fregnani et al. ^10^ conducted a study in the municipality of Barretos (SP) with 1,574 girls aged from 10 to 16 years to evaluate acceptance responses and the VC of a three-dose HPV vaccine. At the end of the third dose, overall VC totaled 85%. To determine the delta of participants who failed to receive the third dose, the authors investigated their parents or guardians’ reasons for refusing vaccination. Overall, 27.4% of responses indicated fear of adverse events; 20.2%, personal reasons; and 14.5%, that the girl refused to receive the vaccine. Other responses pointed to heterogeneous causes such as considering the vaccine unnecessary, distrust of its efficacy, unawareness, and pediatricians or gynecologists’ discouragement of vaccination.

A study conducted in Ohio ^11^ with healthcare providers investigated parents’ rejection of the vaccine, finding that 90% of parents worried about its safety; 79% believed their children or adolescents were sexually inactive; and 63% thought their children would avoid contracting HPV-related diseases, thus evincing that lack of information impacts parents and guardians’ decision regarding vaccination.

### Vaccination Campaigns: World Social Marketing

Australia, England, Mexico, and Peru stood out among all the countries that achieved the WHO-established target of 80% VC in 2018. After documentary surveys, these countries offered information on their vaccination campaigns to prevent HPV. To offer references of good VC practices and the use of social marketing, this study chose informative materials from vaccination campaigns in Australia (considered a reference in vaccination to prevent HPV) and Mexico and Peru (two Latin American countries with sociocultural and economic similarities with Brazil).

Australia offers the vaccine free of charge to girls aged from 11 to 13 years. Despite the fees for boys, the vaccination is approved for all genders from the age of 9 years onward. Australia uses several materials and medical and informational communication aimed at the general population with messages of great impact on health, such as “Cervical Screening saves lives,” “Don’t just sit there,” and “Don’t be ashamed.” This study also found the use of several communication channels, well-timed sequences, frequent dissemination, and information on HPV and the importance of complete vaccination to eradicate and prevent cervical cancer. Such pieces were broadcast on TV, radio, and digital and printed media from 2007 to 2010 and highlighted the strategy of translating and adapting these informative messages for immigrants, such as those from Saudi Arabia, China, and Vietnam.

As part of its vaccination program, Australia has created a national registry of women’s health screenings that dialogues with registered participants via mail, with invitations for testing, reminders, or follow-up. It also created a website with information on HPV and vaccination, several types of communication channels such as an email, postal address, and a free telephone line so women can seek more information and ask questions about cervical cancer issues. It also holds periodic workshops and offers electronic messages (SMS and email) with information about its program, dates, and events. Thus, campaigns increased exams by 15% in 2007 in relation to 2006 and adherence in areas with areas with low examination rates by 21%. Moreover, they obtained an 80.2% third-dose VC in 2017, with a low incidence of nine new cases of cervical cancer for every 100,000 women ^6 , 11 , 12^ .

In Latin America, Mexico had a 97.5% VC for girls aged up to 15 years in 2019. A 2017 local study on the prevalence of HPV in Mexican women showed a 9% rate, of which 77% were type 56, i.e., high risk ^13^ . By treating cervical cancer as a public health problem, the Mexican MH invested in prevention via free vaccination in schools (for girls aged from 9 to 12 years) and in health units for other ages. Teachers mobilize parents, inform them of the date of the vaccination, and request their authorization. The Mexican MH also houses a department dedicated to the health of children and adolescents aged from 10 to 19 years and healthcare providers follow protocol of care to this population that provides for the monitoring and mandatory updating of the vaccination card, including the HPV vaccine. Digital channels distribute vaccination campaign materials and make access to information available to the target audience to engage the community by social marketing.

Peru is another Latin American country that maintained 91% VC in 2019 and incorporated a nonavalent vaccine in its National Immunization Program. Girls aged from 9 to 13 years who are in the fifth grade of primary school are vaccinated in schools for free after their guardians’ consent, who are contacted by government campaigns and schools. Vaccination is administered in three doses (at zero, two, and six months) following the Mexican vaccination schedule. Campaign materials employ accessible language and messages that show the probable outcome of the lack of vaccination, including “A woman dies every five hours from cervical cancer in Peru” ^14^ . Moreover, the Peruvian MH campaign has gifts for girls who are vaccinated. Bracelets and USB sticks in the shape of a heart with the campaign phrase “I got vaccinated. Get vaccinated too” are distributed free of charge to girls who have been vaccinated. The information materials of this vaccination campaign use terms of consent and image resources with characteristics of the local culture and population (strong and striking colors and Peruvian citizens as models).

This evidence and campaign examples enabled the comparison between the social marketing strategies and tactics in Australia, Mexico, and Peru and Brazil. Chart 1 compares countries in the light of social marketing based on the four “P.” The comparison is made between the offered products, carried out promotion, adopted place for vaccination, and vaccine price.

**Chart 1:** Summary of social marketing by country.

Country	Product	Promotion		Place	Price
		Population	Campaigns		
Brazil	Quadrivalent HPV	Girls (9–14 years)	Materials in primary healthcare units	Primary healthcare units	Free
		Boys (11–14 years)			
Mexico	Nonavalent vaccine	Girls (9–12 years) who are in the fifth grade of primary school	Campaigns on TV and radio and printed materials	Schools and primary healthcare units	Free
Australia	Nonavalent vaccine	Boys and girls (9–13 years)	Campaigns on TV and radio and printed materials. National registry for follow-ups, websites, and a telephone line	Schools and primary healthcare units	Free for individuals aged from 11 to 13 years
Peru	Nonavalent vaccine	Girls (9–13 years) who are in the fifth grade of primary school	Campaigns on TV and radio and printed materials	Schools	Free
Barretos Study	Quadrivalent HPV	Girls (10–16 years)	Campaigns on TV and radio and printed materials	Schools and hospitals	Free

HPV: human papillomavirus

### The Increase in HPV Vaccine Coverage in Brazil: Application of Quality Tools

**Figure 1:**
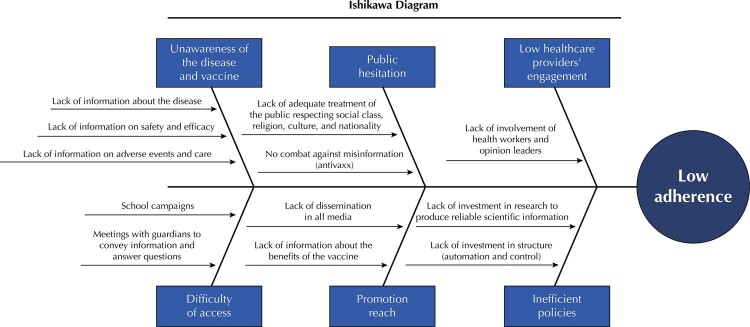
Ishikawa diagram.

**Figure 2:**
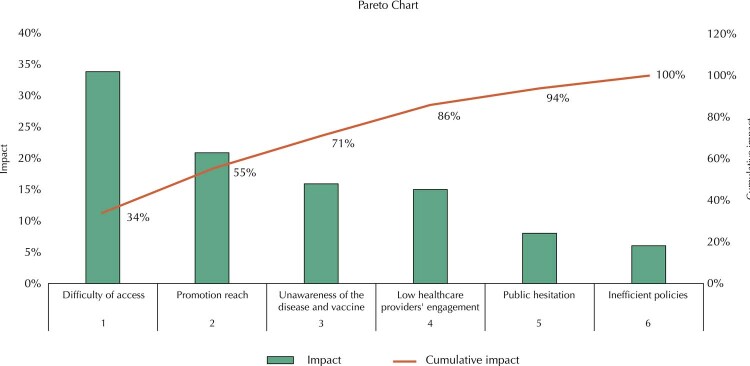
Pareto chart.

The chosen quality tools offer different forms of analyzing results. The Ishikawa diagram ( Figure 1 ) shows the six main causes and subcauses of low adherence. The Pareto graph ( Figure 2 ) shows the impact of these causes in an isolated and cumulative way. 5W2H ( Chart 2 ) describes the main mitigation proposals for the identified causes to reduce their quantified impact.

In the Pareto chart, the bars from the left to right show the problems that most impact adherence to Brazilian vaccination campaigns. The red line shows the cumulative percentage of the impact on vaccination campaigns. Thus, it is possible to observe that causes 1 and 2 have a cumulative impact of 55%. It can be inferred that focusing attention on the first two causes could solve the main problems that have a 55% impact on adherence increase, which fails to mean that coverage can increase by as much as 55% since other problems can still impacts results, but a significant increase in coverage of over two digits may be expected.

**Chart 2:** 5W2H tool.

What	Why	Where	When	Who	How	How much
Disseminate information about vaccine safety and efficacy	Information on safety and efficacy conveys confidence about the vaccine to the public and facilitates decision-making by increasing adherence. This information is the main target of anti-vaccine groups.	Throughout the Brazilian territory	During the campaign in both doses	Federal and state governments	Dissemination on TV, internet, radios, billboards, schools, primary healthcare units, and by physicians and healthcare providers.	NA
Inform the vaccination scheme	This information helps the target audience plan itself regarding deadlines and procedures.	Throughout the Brazilian territory	During the campaign in both doses	Federal and state governments	Dissemination on TV, internet, radios, billboards, schools, primary healthcare units, and by physicians and healthcare providers.	NA
Disseminate expected adverse symptoms and what should be done in such cases	Information about expected symptoms prevents panic and conveys tranquility and information on safety and efficacy, educating the public on the procedure to be followed and avoiding unnecessary searches for physicians and hospitals in case of events.	Throughout the Brazilian territory	During the campaign in both doses	Federal and state governments	Dissemination on TV, internet, radios, billboards, schools, primary healthcare units, and by physicians and healthcare providers.	NA
Inform the gratuity of the vaccine	This information is well known to Brazilians, but it is necessary to consider the immigrants who live in it. Strengthen information on gratuity will remove another possible barrier to adherence.	Throughout the Brazilian territory	During the campaign in both doses	Federal and state governments	Dissemination on TV, internet, radios, billboards, schools, primary healthcare units, and by physicians and healthcare providers.	NA
Inform campaign investments	Information about investment brings a sense of responsibility to some people and reinforces the importance of transparent spending.	Throughout the Brazilian territory	During the campaign in both doses	Federal and state governments	Dissemination on TV, internet, and radios.	NA
Using schools for vaccination	Vaccination in schools facilitates access to children and guardians. It facilitates adherence, information dissemination, and campaign control.	Schools throughout Brazil	During the campaign in both doses	Federal and state governments	Create of a program with shared responsibilities between the federal and state governments.	NA
Using primary healthcare units for repechage	For audiences outside the goal of the campaign, primary healthcare units should remain a primary vaccination site. For children who missed vaccination in schools for some reason, these institutions will serve as a repechage site.	Primary healthcare units	During the campaign in both doses	Federal and state governments	Use the Unified Health System (SUS).	NA
Prepare and distribute information leaflets	Leaflets help to disseminate information about the vaccine and serve as a manual to ask questions and know what to do in case of events, etc.	Schools, primary healthcare units, and with physicians and healthcare providers	During the campaign in both doses	Federal and state governments	Create campaign materials with the help of physicians, healthcare providers, and advertising agencies and distributing it to the target audience and guardians by schools, primary healthcare unit, and physicians.	NA
Giving lectures to guardians and the target audience in schools	As schools facilitate access to guardians, children, and adolescents, it is possible to directly transmit information and solve doubts in a personalized way.	Schools	During the campaign in both doses	Federal and state governments	Send prepared healthcare providers to lecture to guardians, children, and adolescents.	NA
Identify the target audience and educate the age group	It is important that the target audience is clearly identified and informed so that they know they will be approached or should seek help.	Schools	During the campaign in both doses	Federal and state governments	By physicians and healthcare providers using an updated IBGE database.	NA
Tailor the information to different audiences	An important manner of inclusion, in which people are approached with appropriate information, respecting the culture, religion, and nationality of all.	In all campaign materials	During the campaign in both doses	Federal and state governments	Using advertising agencies.	NA
Using mass media	The internet, TV, and radio are the main global communication channels and Brazil is no different. To favor any to disseminate campaign information excludes a large part of the population without access to information.	TV, internet, radios, and billboards	During the campaign in both doses	Federal and state governments	Using pieces, commercials, jingles, and influencers.	NA
Focus on information about the benefits of the vaccine	Information on the lack of vaccination (diseases, risks, and harms) should be avoided. People absorb this information as negative points of the vaccine.	In all campaign materials	During the campaign in both doses	Federal and state governments	Creating campaign materials with the help of physicians, healthcare providers, and advertising agencies.	NA
Include information on pap smears in the campaign	Although the percentage of pap smears is high ^15^ , it will be possible to further increase adherence by mass dissemination.	In all campaign materials	During the campaign in both doses	Federal and state governments	Creating campaign materials with the help of physicians, healthcare providers, and advertising agencies.	NA
Encourage medical prescription	Use federal and regional boards to give physicians directives about the vaccine.	Federal and regional councils	All the time (should become a daily routine)	Federal and state governments	Contact the health councils in Brazil and start a program to create guidelines.	NA
Prepare physicians and healthcare providers to answer questions.	Send support materials via federal and regional councils to solve the public’s doubts, including guardians.	Federal and regional councils	All the time (should become a daily routine)	Federal and state governments	Contact the health councils in Brazil and start a program to create guidelines.	NA
Automate notaries for automatic registration on ConectSus	The idea is that at the time of registration of a newborn, the notary can automatically create a registration for the child in ConectSus or generate an access code that enables the system to control their vaccination since birth.	Notaries and application	All the time (should become a daily routine)	Federal and state governments	Create a function to interconnect notary systems with ConectSus.	NA
Fight disinformation (antivaxx)	These radical groups are growing rapidly and the internet has facilitated the spread of false information. They must be fought before the issue becomes even more serious.	Throughout the Brazilian territory	All the time	Federal and state governments	Disinformation is fought with reliable information, which is only possible with investment in research to generate knowledge.	NA
Investment in research	It is impossible to move forward as a society and as a country if investments in research are suppressed. Research generates knowledge and relevant information that determine how the country should react to a given problem. Brazil has an investment rate far below what is necessary to generate relevant knowledge for society.	Throughout the national territory (universities, hospitals, schools, research centers, etc.)	Must be part of the annual budget	Federal and state governments	The state and federal governments should increase their budget for investments in research. Considering the current numbers, it is practically impossible to significantly advance in the following years ^16^ .	NA

NA: Not applicable

The Ishikawa diagram evinces the unawareness of HPV and its vaccine, public hesitancy, difficulty of access, scope of promotion, healthcare providers’ low engagement, and inefficient policies constitute the main causes of low adherence to the vaccination campaign in Brazil. This study used a Pareto graph to quantify such causes. It numerically shows which should be prioritized by action plans. To determine the degree of impact, this study used references from research ^10 , 11 , 17^ that estimated each raised point on the X axis of our Pareto chart. After classifying the causes and quantifying their impact, it was possible to elaborate the results of a third quality tool to suggest proposals for increasing VC. The bars from the left to right in the Pareto chart show the problems that most impact adherence to Brazilian vaccination campaigns. The red line shows the cumulative percentage of their impact on vaccination campaigns. Thus, it is possible to observe that causes 1 and 2 have a 55% cumulative impact, suggesting that the focus applied to the first two causes could solve the main problems that impact adherence increase by 55%.

Chart 2 shows the proposed action plan according to 5W2H, which identifies what should be done, why, where, when, by whom, how, and how much it would cost. The tool can also transform each activity into controllable and measurable metrics. This study ignored activity costs as their estimation would require diverse and complex information on public health.

## DISCUSSION

Australia, Mexico, and Peru showed good application and integration of Kotler’s four conceptual “P” as the foundations of social marketing strategies ^7 , 8^ . They correctly identified their target audience, free products facilitated access for the population, and the environment of application and information to health (schools) and the promotion tactics reflected the need for objective communication and easy acceptance by the target audience in each country ^14 , 18 , 20^ . Moreover, campaign material customization included an accessible language to immigrants in these countries (as with Australia) without judging people by their creeds, tastes, and cultures.

Regarding the quality tools with social impact, the mapping of the main causes is expected to enable the creation of plans for each of them and solve low adherence. Each cause significantly impacts the target audience’s adherence to vaccination campaigns. Predicting this impact and taking assertive measures contributes to increasing population engagement, creating collective awareness, and raising VC. The Pareto graph also shows the percentage of isolated impact for each cause. A 2014 study on how social marketing impacted vaccination campaigns ^18^ estimated a 34% difficulty of access, i.e., social marketing can increase vaccination adherence by 34%. This datum is corroborated by the 35% increase in coverage in Uruguay after it changed its vaccination strategy to schools.

The Australian survey evaluated promotion scope, which increased adherence up to 21% ^12^ . A study conducted in Mexico ^19^ estimated unawareness about the vaccine at about 16%. Daley et al. ^20^ state that healthcare providers’ recommendations and prescriptions can increase adherence by 15%.

According to data from the Barretos study, about 8% of the target audience show vaccine hesitancy, leaving the rest of the impact on inefficient policies (6%) ^10^ .

## CONCLUSION

The consecutive stages of our bibliographic and documentary review, the determination of the actual VC (%) in Brazil, and the comparison of the use of social marketing strategies in campaigns in countries with high VCs was essential to apply quality tools that classified and quantified the causes of low VC in Brazil. Finally, the proposal for an action plan (detailed in the 5W2H tool) may serve to build a potential social document with proposals based on global good practices that can direct the Brazilian MH to follow more effective strategies to achieve the VC goal recommended by the WHO.
