# Supplementary material for: HPV immunization in Brazil and proposals to increase adherence to vaccination campaigns
Source: Rev Saude Publica. 2023 Oct 24;57:79. doi: 10.11606/s1518-8787.2023057005410 (PMC10609638; doi:10.11606/s1518-8787.2023057005410)
Supplement: Translation

# Translation: Imunização do HPV no Brasil e propostas para aumento da adesão à campanha de vacinação

**Authors:** Wagner Mesojedovas Santos, Debora Mesojedovas Santos, Márcia Santana Fernandes

**Affiliations:** I Brasil Universidade Federal do Rio Grande do Sul. Hospital de Clínicas de Porto Alegre. Programa de Pós-graduação em Pesquisa Clínica. Porto Alegre, RS, Brasil; II Brasil Universidade Federal do Rio Grande do Sul. Hospital de Clínicas de Porto Alegre. Laboratório de Pesquisa de Bioética e Ética na Ciência. Porto Alegre, RS, Brasil

**Keywords:** Papilomavírus Humano, Neoplasias do Colo do Útero, Marketing Social, Imunização, Programas de Imunização

## Abstract

**OBJETIVOS:**

Identificar as possíveis causas da baixa adesão à campanha de vacinação no Brasil, identificar e analisar as campanhas relacionadas ao papilomavírus humano (HPV) no Brasil e em outras regiões do mundo, e aplicar ferramentas de qualidade para elaboração de propostas para aumento da cobertura vacinal (CV) para prevenção do HPV no país.

**MÉTODO:**

Pesquisa de natureza qualitativa e dedutiva-hipotética. A técnica utilizada para o desenvolvimento do método é a revisão narrativa da literatura, em particular estudando as narrativas e formatos aplicados nas campanhas de vacinação no Brasil.

**RESULTADOS:**

Identificou-se que, em 2019, a CV no Brasil foi de 49,6%, diferentemente de países como Austrália (80,2% em 2017), México (97,5% em 2019) e Peru (91% em 2019). Evidências do uso de estratégias de marketing social para engajamento comunitário foram encontradas nas campanhas de vacinação utilizadas como boas práticas nesses países.

**CONCLUSÃO:**

Com as informações encontradas foram aplicadas três ferramentas de qualidade (diagrama de Ishikawa, gráfico de Pareto e 5W2H) que classificaram e quantificaram as causas da baixa CV no Brasil e, com isso, viabilizaram propostas que podem direcionar o MS à tomada de estratégias mais eficazes para atingimento da meta de CV do HPV recomendada pela OMS.

## INTRODUÇÃO

A principal via de transmissão do papilomavírus humano (HPV) é o contato sexual. Dentre os mais de 100 tipos de HPV reportados na literatura médica, 50 tipos acometem a mucosa do aparelho genital e pelo menos 15 são classificados como de alto risco, ou seja, com potencial carcinogênico^1^. O HPV de alto risco é responsável por 99% dos casos de câncer de colo de útero, sendo o tipo 16 o mais comum detectado no carcinoma^1^, e está dentre os quatro tipos de cânceres que mais matam por ano no Brasil (6.526 óbitos em 2019)^2^. No mundo, em 2018, foram 311 mil vítimas fatais^3^. Dentre todos os tipos de câncer, o de colo de útero é o único que atualmente conta com um imunizante para prevenção.

Os tipos de HPV 16 e 18 são responsáveis por cerca de 70% dos casos de câncer de colo do útero; os tipos 6 e 11 são responsáveis por mais de 90% dos casos de verrugas genitais^4^. Esses quatro tipos são alvos profiláticos da vacina quadrivalente recombinante para a prevenção do HPV distribuída desde 2014 no Sistema Único de Saúde (SUS) brasileiro por meio do Programa Nacional de Imunização (PNI) e tem meta de cobertura vacinal (CV) de 80%, alinhada à Organização Mundial da Saúde (OMS). Entretanto, após sete anos do início da campanha nacional de vacinação para prevenção do HPV (2014–2021), os dados do Ministério da Saúde (MS) mostram que a meta não foi atingida no país^5^.

Globalmente, campanhas de vacinação para prevenção do HPV foram adotadas por diversos países em seus sistemas de saúde, seguindo as metas e recomendações da OMS. Atualmente, 114 países incluíram no calendário vacinal duas ou três doses em seus programas de saúde pública^3^. Embora a OMS preconize a cobertura de 80% de vacinação do público-alvo para todos os países, alguns deles apresentam CV abaixo da meta recomendada, como é o caso de Irlanda, com 73% de cobertura, e Suíça, com 58,9%. Em direção oposta, países como Austrália, México e Peru apresentam CV acima da meta recomendada pela OMS^6^. No geral, é possível observar em diferentes canais de comunicação e na literatura que tais países utilizaram em suas campanhas de vacinação técnicas de marketing social para melhor engajamento do público-alvo.

O marketing social é um conjunto de iniciativas com finalidade sociais cujo objetivo principal é, através de diferentes abordagens, modificar o comportamento e influenciar questões sociais, sem foco na comercialização de bens ou serviços^7^. O grande ponto defendido por Kotler, idealizador do marketing social, é que não é possível influenciar o comportamento das pessoas sem levar em consideração o aspecto socioeconômico. Além disso, o marketing social defende que a capacidade de gerar insumos e ferramentas necessárias para desenvolver intervenções eficazes é de fundamental importância, deixando claro que o foco é o bem-estar do indivíduo, grupo ou sociedade. A aplicação do marketing social pode ser feita com base nos quatro “P” defendidos por Kotler ( *product, price, place and promotion* : produto, preço, local e promoção, respectivamente). Quando aplicados em conjunto, os quatro “P” fornecem uma espécie de guia para o sucesso de uma estratégia de marketing social e são considerados o coração do marketing social por Nowak^8^. Logo, infere-se que países com metas de vacinação muito abaixo da recomendada pela OMS, como o Brasil, por exemplo, não estão utilizando os benefícios do marketing social. Dentre os benefícios, destacam-se quatro que têm ligação direta com os quatro “P”: a oferta de benefícios reais, custos, facilidade de acesso e mensagem personalizada.

Ainda assim, no contexto de marketing social aplicado por Austrália, México e Peru em suas campanhas de vacinação, considera-se a oportunidade de utilizar ferramentas de qualidade que possam identificar problemas, causas e possíveis soluções para melhorar a CV no Brasil. As ferramentas de qualidade são instrumentos que visam facilitar diversas atividades de uma corporação para melhor gestão de seus processos^9^.

Um desses instrumentos é o diagrama de Ishikawa, uma ferramenta de qualidade utilizada para gestão de processos em que é necessário mapear as entregas de produtos ou serviços dentro de parâmetros definidos. Também conhecido como causa e efeito ou diagrama de espinha de peixe, o diagrama de Ishikawa é basicamente uma ferramenta visual que auxilia em análises de problemas^9^e permite realizar a identificação rápida da causa raiz dos problemas para mitigá-los.

Outra ferramenta de qualidade é o gráfico de Pareto, que possibilita priorizar os problemas e atacá-los de forma a obter resultados significativos de forma rápida, sem a necessidade de mitigar todas as causas de uma só vez. O gráfico de Pareto é conhecido como gráfico 80-20, pois se baseia na regra que diz que 80% dos resultados advém de 20% das ações realizadas. Com isso é possível focar as ações em 20% dos principais problemas para conseguir 80% de resultado^9^.

Por fim, cita-se a ferramenta 5W2H, uma técnica simples que permite guiar as pessoas em situações e problemas gerando um plano de ação para cada problema identificado. A ferramenta traz sete questões, criando um roteiro que permite entender o problema e seu impacto para saber como resolvê-lo. As perguntas são: *o quê?, por quê?, onde?, quando?, quem?, como?* e *quanto custa?* ^9^.

A presente revisão da literatura teve como objetivo 1) identificar as possíveis causas do não alcance da meta de 80% de imunização no Brasil. Em paralelo, buscou 2) listar as principais estratégias e táticas de campanhas de adesão à vacinação para prevenção do HPV realizadas em outros países para servir como boas práticas de adesão no Brasil com base na abordagem do marketing social. Por fim, este trabalho objetivou 3) aplicar ferramentas de qualidade que classificaram e quantificaram as causas da baixa CV e, com isso, viabilizaram propostas que podem direcionar o MS do Brasil na tomada de estratégias mais eficazes a fim de atingir a meta de CV recomendada pela OMS.

## MÉTODOS

Para 1) identificação das causas do não alcance da CV no Brasil, a pesquisa bibliográfica e documental foi o método adotado na coleta das informações para as análises sobre as campanhas de vacinação realizadas no Brasil e no mundo utilizando websites de domínio público. Também foi realizada uma revisão narrativa da literatura médica na qual foram usadas palavras-chave como “papilomavírus humano”, “vacinação” e “campanha de vacinação HPV” em bases de dados como PubMed, Embase, Scopus, Bireme e SciELO (jan./2010–dez./2022).

Para coleta das informações e dados da campanha de vacinação no Brasil (2014–2021), foram utilizados dados oficiais disponíveis no site do MS (https://falabr.cgu.gov.br/), na aba “acesso à informação”. Também se incluíram dados públicos disponíveis no portal PNI (http://pni.datasus.gov.br/) e informações complementares de 2019, acessadas via website da Sociedade Brasileira de Oncologia Clínica (SBOC). Os dados foram separados de acordo com a cobertura para a primeira e segunda dose da vacina, e foi calculada a média simples para ambos os sexos para avaliar a cobertura geral e possibilitar a avaliação da cobertura em relação à meta preconizada pelo próprio MS.

Para 2) identificar com base no marketing social as principais estratégias e táticas de campanhas de adesão à vacinação realizadas globalmente, foi necessário levantar os dados de CV de outros países, obtidos no relatório oficial de indicadores emitido pela OMS (The Global Health Observatory)^3^. Esses dados foram avaliados por meio de trabalhos publicados, sendo selecionados três países cujas informações das campanhas de vacinação foram divulgadas e que conseguiram manter a meta preconizada pela OMS para eficácia da imunização na intenção de erradicar o câncer, para fins de comparação. Com base nas informações obtidas de CV e campanhas utilizadas, foi feita uma avaliação para tentar identificar os possíveis problemas na campanha de vacinação do HPV no Brasil. O marketing social utilizado pelo MS foi avaliado de acordo com a teoria preconizada e defendida por Philip Kotler^7^e com estratégias utilizadas em trabalhos publicados.

Por fim, 3) foi feita a análise de conteúdo de todo o material organizado para traduzi-lo em resultados interpretativos utilizando as ferramentas de qualidade diagrama de Ishikawa, gráfico de Pareto e ferramenta 5W2H^9^, para melhor exposição das informações.

## RESULTADOS

### Cobertura da Vacina Contra HPV no Brasil

Com base nos dados fornecidos pelo MS, PNI e SBOC, determinou-se a CV de 49,6% no Brasil ( Tabela ). Os dados fornecidos pelo MS consideram o período de 2014 a 2020 em valor absoluto, sem estratificar a cobertura anual. Para os anos de 2014 e 2015 o esquema de vacinação adotado pelo MS era de três doses com intervalo de 0, 2 e 6 meses entre elas. A partir de 2016 o esquema foi alterado para duas doses com intervalo mínimo de 6 meses entre elas. O público-alvo definido para o início do programa em 2014 foram meninas com idade de 11 a 14 anos. O público foi alterado em 2015 com a inclusão dos meninos no programa e o limite inferior foi estendido para 9 anos de idade, o qual permanece até hoje.

**Tabela:** Cobertura vacinal com a primeira e a segunda dose (D1 e D2) da vacina contra HPV quadrivalente, na população masculina e feminina de 9 a 14 anos, Brasil de 2014 a 2020.

Sexo	População-alvo	D1(%)	D2 (%)	Média (%)
Feminino	9–14 anos	82,1	54,5	68,3
Masculino	9–14 anos	38,6	23,5	31
Média total		60,3	39	49,6

Fonte: SIPNI TABNET BD – MS (janeiro/21). HPV: papilomavirus humano; D1: primeira dose; D2: 2ª dose.

Para cálculo da CV em meninas, o período considerado foi de março de 2014 a novembro de 2020. Para meninos, considerou-se o período de março de 2015 a novembro de 2020.

### As Razões para Hesitação e Recusas à Vacina Contra HPV

Na revisão bibliográfica, documental e da literatura médica, foram selecionadas duas publicações de vida real que aplicaram questionários aos responsáveis quando eles se recusavam vacinar as crianças e adolescentes.

Em setembro de 2010, Fregnani et al.^10^iniciaram um estudo na cidade de Barretos (SP) envolvendo 1.574 meninas entre 10 e 16 anos de idade com o objetivo de avaliar a resposta de aceitação e a CV das três doses da vacina contra HPV. Ao final da 3ª dose, a CV geral foi de 85%. Para determinar o delta de participantes que não completaram a 3ª dose, investigaram-se junto aos seus pais ou responsáveis legais as razões pelas quais a vacinação foi recusada. No geral, 27,4% das respostas apontaram medo dos eventos adversos da vacina; 20,2% razões pessoais; e 14,5% afirmaram que a menina não desejava se vacinar. As demais respostas apontaram causas heterogêneas como a vacina não ser necessária, não confiar na eficácia da vacina, não conhecer a vacina, e o desaconselhamento da vacinação pelo médico pediatra ou ginecologista.

Um outro estudo realizado em Ohio^11^com profissionais de saúde investigou a percepção para a recusa dos pais em relação à vacina. A pesquisa apontou que 90% dos pais estão preocupados com a segurança da vacina; 79% acreditam que a criança ou adolescente não é sexualmente ativo; 63% acreditam que os filhos não irão contrair doenças relacionadas ao HPV. Logo, evidencia-se que a falta de informação impacta a decisão dos pais e responsáveis em relação à vacinação.

### Campanhas de Vacinação: Marketing Social Mundial

De todos os países que em 2018 se mantiveram dentro da meta de 80% de CV estabelecida pela OMS e que, após o levantamento documental, apresentavam informações sobre as suas campanhas de vacinação para prevenção do HPV, destacaram-se Austrália, Inglaterra, México e Peru. Para fins referenciais de boas práticas de CV e da utilização do marketing social, foram selecionados materiais informativos das campanhas de vacinação da Austrália (país considerado referência na vacinação para prevenção do HPV) e de dois países latinos, México e Peru (países que têm similaridades socioculturais e econômicas com o Brasil).

Na Austrália, a vacina é administrada gratuitamente em meninas de 11 a 13 anos. Meninos não recebem a vacina gratuitamente, mas ela é aprovada para ambos os sexos a partir dos 9 anos. A Austrália utiliza diferentes materiais e peças de comunicação médica e informativa direcionados à população geral com mensagens de alto valor de impacto à saúde, como “O [exame] Papanicolau pode salvar sua vida”, “Não fique só aí sentado”, “Não fique com vergonha”. Além disso, notou-se o uso de diversos canais de comunicação e sequência e frequência bem ritmadas na periodicidade da divulgação das mensagens e informações sobre o HPV e a importância da vacinação completa para sua erradicação e prevenção do câncer de colo de útero. As peças foram veiculadas na TV, rádio, mídia digital e material impresso durante os anos de 2007 a 2010, destacando-se a estratégia de traduzir e adaptar as mensagens informativas para imigrantes, como Arábia Saudita, China e Vietnã.

Como parte do programa de vacinação, o governo criou um registro nacional de exames de saúde da mulher, cuja comunicação com as participantes registradas é feita via correio postal, com cartas de convite para realização de exames, lembretes ou acompanhamento. Também foi criado um website com informações sobre o HPV e vacinação, com diversos tipos de canais de comunicação como e-mail, endereço postal e telefone para chamadas gratuitas, para as mulheres poderem buscar mais informações e tirar dúvidas sobre qualquer questão relacionada ao câncer do colo de útero. Ainda há workshops periódicos e mensagens eletrônicas (SMS e e-mail) com informações sobre o programa, datas e eventos. Como resultado, as campanhas foram responsáveis pelo aumento de 15% de exames em 2007 em relação ao ano anterior e, nas áreas em que a adesão aos exames era baixa, houve aumento de 21%. Além disso, em 2017, a CV da 3ª dose era de 80,2%, com uma incidência baixa de nove novos casos de câncer de colo de útero para cada 100 mil mulheres^6 , 11 , 12^.

No contexto da América Latina, em 2019, o México apresentou uma CV de 97,5% para meninas até 15 anos de idade. Em 2017, foi publicado um estudo local sobre a prevalência de HPV nas mulheres mexicanas e o resultado foi de 9%. O estudo também mostrou que 77% eram do tipo 56, de alto risco^13^. Ao tratar o câncer de colo do útero como problema de saúde pública, o MS mexicano investiu na prevenção por meio da vacinação gratuita feita nas escolas (para meninas de 9 a 12 anos) e nas unidades de saúde para as demais idades. Os pais são acionados por professores e informados sobre quando a vacinação irá ocorrer, mediante a sua autorização. Além disso, o MS mexicano abriga um departamento dedicado especificamente à saúde de crianças e adolescentes com idade entre 10 e 19 anos, cujo protocolo de atendimento dos profissionais de saúde a essa população prevê a observância e atualização obrigatória da carteira de vacinação, incluindo a vacina contra HPV. Materiais de campanha de vacinação são distribuídos e o acesso à informação é disponibilizado ao público-alvo da vacina nos canais digitais, visando engajamento comunitário por meio do marketing social.

O Peru é outro país latino-americano que manteve 91% de CV em 2019. O país incorporou em seu PNI a vacina nonavalente. As meninas com idade entre 9 e 13 anos que estão na 5ª série do primário são vacinadas nas escolas de forma gratuita após o consentimento dos responsáveis, que são alcançados por meio de campanhas do governo e pelas próprias escolas. A vacinação é administrada em três doses (0, 2 e 6 meses) seguindo o esquema vacinal do México. Em relação aos materiais de campanha, observou-se o uso de linguagem de fácil acesso e com mensagens que demonstram o provável desfecho da falta de vacinação, como: “Uma mulher morre a cada cinco horas em decorrência do câncer de colo do útero no Peru”^14^. Além disso, a campanha do MS do Peru conta com brindes para as meninas que são vacinadas. Pulseiras e *pendrives* no formato de coração com a frase da campanha “Eu me vacinei. Vacine-se você também” são distribuídos gratuitamente para as meninas que se vacinaram. Os materiais informativos das campanhas de vacinação utilizam termos de consentimento e recursos de imagens com características da cultura e população local (cores fortes e chamativas; cidadãs peruanas como modelos nos materiais de campanha).

Com as evidências e exemplos de suas campanhas, foi possível comparar as estratégias e táticas de marketing social utilizadas pela Austrália, México e Peru em relação ao Brasil. O Quadro 1 faz um comparativo entre os países à luz do marketing social com base nos quatro “P”. A comparação é feita entre os produtos oferecidos, a promoção realizada, o local adotado para a vacinação e o preço da vacina.

**Quadro 1:** Resumo do marketing social por país.

País	Produto	Promoção		Local	Preço
		População	Campanhas		
Brasil	Vacina quadrivalente	Meninas (9–14 anos)	Materiais fixados nos postos de saúde	Postos de saúde	Gratuita
		Meninos (11–14 anos)			
México	Vacina nonavalente	Meninas (9–12 anos) que estão na 5º série do ensino primário	Campanhas na TV, rádio e material impresso	Escolas e postos de saúde	Gratuita
Austrália	Vacina nonavalente	Meninas e meninos (9–13 anos)	Campanhas na TV, rádio, mídia digital e material impresso. Registro nacional para acompanhamento, sites e telefone	Escolas e postos de saúde	Gratuita entre 11 e 13 anos
Peru	Vacina nonavalente	Meninas (9–13 anos) que estão na 5º série do ensino primário	Campanhas na TV, rádio, material impresso e brindes	Escolas	Gratuita
Estudo Barretos	Vacina quadrivalente	Meninas (10–16 anos)	Campanhas na TV, rádio, material impresso	Escolas e hospital	Gratuita

### Aumento da Cobertura da Vacina contra HPV no Brasil: Aplicações das Ferramentas de Qualidade

As ferramentas de qualidade escolhidas trazem diferentes formas de análise dos resultados. O diagrama de Ishikawa ( Figura 1 ) mostra as seis principais causas e subcausas da baixa adesão. O gráfico de Pareto ( Figura 2 ) mostra o impacto dessas causas de forma isolada e acumulada. A ferramenta 5W2H ( Quadro 2 ) apresenta as principais propostas de mitigação para as causas identificadas visando a redução do impacto quantificado.

**Figura 1:** Diagrama de Ishikawa.

**Figura 2:** Gráfico de Pareto.

Nota: as barras a partir da esquerda mostram os problemas que mais impactam a adesão à campanha de vacinação contra HPV no Brasil. A linha vermelha mostra o percentual acumulado do impacto na campanha de vacinação. Dessa forma, é possível observar que as causas 1 e 2 têm impacto acumulado de 55%. Infere-se que o foco aplicado às atenções nas duas primeiras causas poderia resolver os principais problemas que impactam em 55% o aumento da adesão. Isso não significa que a cobertura poderá aumentar em até 55%, uma vez que os outros problemas ainda podem impactar o resultado, mas espera-se aumento significativo da cobertura acima de dois dígitos.

**Quadro 2:** Ferramenta 5W2H.

O que	Por que	Onde	Quando	Quem	Como	Quanto
Divulgar informações sobre segurança e eficácia da vacina	As informações sobre a segurança e eficácia transmitem ao público confiança sobre a vacina e facilitam a tomada de decisão aumentando a adesão. Essas informações são os principais alvos dos grupos antivacina.	Em todo o território brasileiro	Durante o período de campanha. Nas duas doses.	Governo federal e governos estaduais	Divulgação na TV, internet, rádios, outdoors, escolas, postos de saúde e por médicos e profissionais da saúde.	NA
Informar o esquema de vacinação	Essa informação ajuda no planejamento do público-alvo em relação a prazos e procedimentos.	Em todo o território brasileiro	Durante o período de campanha. Nas duas doses.	Governo federal e governos estaduais	Divulgação na TV, internet, rádios, outdoors, escolas, postos de saúde e por médicos e profissionais da saúde.	NA
Divulgar os sintomas adversos esperados e o que deve ser feito em caso de eventos	As informações sobre os sintomas esperados evita o pânico e juntamente com as informações de segurança e eficácia transmitem tranquilidade, além de informar ao público o procedimento a ser seguido, evitando assim uma busca desnecessária por médicos e hospitais em caso de eventos.	Em todo o território brasileiro	Durante o período de campanha. Nas duas doses.	Governo federal e governos estaduais	Divulgação na TV, internet, rádios, outdoors, escolas, postos de saúde e por médicos e profissionais da saúde.	NA
Informar a gratuidade da vacina	Essa é uma informação bastante conhecida dos brasileiros, porém é preciso considerar os imigrantes que vivem no país. Reforçar a informação sobre a gratuidade eliminará mais uma possível barreira para a adesão.	Em todo o território brasileiro	Durante o período de campanha. Nas duas doses.	Governo federal e governos estaduais	Divulgação na TV, internet, rádios, outdoors, escolas, postos de saúde e por médicos e profissionais da saúde.	NA
Informar o investimento em campanha	A informação sobre o investimento traz um senso de responsabilidade para algumas pessoas além de ser importante a transparência em relação aos gastos.	Em todo o território brasileiro	Durante o período de campanha. Nas duas doses.	Governo federal e governos estaduais	Divulgação na TV, internet e rádios.	NA
Utilizar as escolas para a vacinação	A vacinação nas escolas permite fácil acesso as crianças e responsáveis. Facilita a adesão a divulgação de informações e o controle sobre a campanha.	Escolas de todo o Brasil	Durante o período de campanha. Nas duas doses.	Governo federal e governos estaduais	Criação de um programa com responsabilidades compartilhadas entre o governo federal e estadual.	NA
Utilizar os postos de saúde para uma repescagem	Para os demais públicos que não fazem parte da meta da campanha, deve se manter os postos de saúde como um local primário de vacinação, e para as crianças que perderam a vacinação nas escolas por alguma razão, os postos servirão como local de repescagem.	Postos de saúde	Durante o período de campanha. Nas duas doses.	Governo federal e governos estaduais	Utilizando o Sistema Único de Saúde (SUS).	NA
Preparar e distribuir folhetos informativos	Os folhetos auxiliam na divulgação das informações sobre a vacina. Também servem como um manual para tirar dúvidas e saber o que fazer em caso de eventos etc.	Escolas, postos de saúde e com médicos e profissionais da saúde	Durante o período de campanha. Nas duas doses.	Governo federal e governos estaduais	Criando materiais de campanha com auxílio de médicos, profissionais da saúde e agências de publicidades e distribuindo para o público-alvo e responsáveis mediante escolas, postos e médicos.	NA
Dar palestras nas escolas para os responsáveis e público-alvo	Como as escolas facilitam o acesso aos responsáveis e às crianças e adolescentes, é possível transmitir as informações de forma direta e sanar todas as dúvidas de forma personalizada.	Escolas	Durante o período de campanha. Nas duas doses.	Governo federal e governos estaduais	Enviar profissionais de saúde preparados para palestrar para os responsáveis, crianças e adolescentes.	NA
Identificar o público-alvo e informar a faixa etária	É importante que o público-alvo seja identificado e informado de forma clara para que saibam que serão abordados ou devem procurar auxílio.	Escolas	Durante o período de campanha. Nas duas doses.	Governo federal e governos estaduais	Por médicos e profissionais de saúde, utilizando uma base de dados atualizada do IBGE.	NA
Adequar as informações para os diferentes públicos	Uma forma de inclusão importante, na qual as pessoas são abordadas com informações adequadas, respeitando a cultura, a religião e nacionalidade de cada um.	Em todos os materiais de campanha	Durante o período de campanha. Nas duas doses.	Governo federal e governos estaduais	Utilizando agências de publicidade.	NA
Utilizar os meios de comunicação em massa	A internet, a TV e o rádio são os principais canais de comunicação globais e no Brasil não é diferente. Preterir qualquer um deles para divulgação das informações da campanha significa excluir grande parte da população sem acesso à informação.	TV, internet, rádios e outdoors	Durante o período de campanha. Nas duas doses.	Governo federal e governos estaduais	Utilizando peças, comerciais, jingles e influenciadores.	NA
Focar nas informações sobre os benefícios da vacina	As informações relacionadas à falta de vacinação (doenças, riscos e malefícios) devem ser evitadas. As pessoas absorvem essas informações como pontos negativos da vacina.	Em todos os materiais de campanha	Durante o período de campanha. Nas duas doses.	Governo federal e governos estaduais	Criando materiais de campanha com auxílio de médicos, profissionais da saúde e agências de publicidades.	NA
Incluir informações para o exame de Papanicolau na campanha	Embora o percentual de exame de Papanicolau esteja alto^15^será possível aumentar ainda mais a adesão ao fazer a divulgação em massa.	Em todos os materiais de campanha	Durante o período de campanha. Nas duas doses.	Governo federal e governos estaduais	Criando materiais de campanha com auxílio de médicos, profissionais da saúde e agências de publicidades.	NA
Incentivar a prescrição médica	Utilizar os conselhos federais e regionais para dar diretivas aos médicos sobre a vacina.	Conselhos federais e regionais	O tempo todo (deve se tornar rotina diária)	Governo federal e governos estaduais	Contatar os conselhos de saúde do Brasil e iniciar um programa para criar diretrizes.	NA
Preparar médicos e profissionais de saúde para tirar dúvidas.	Enviar por meio dos conselhos federais e regionais materiais de apoio para sanar dúvidas do público, incluindo os responsáveis.	Conselhos federais e regionais	O tempo todo (deve se tornar rotina diária)	Governo federal e governos estaduais	Contatar os conselhos de saúde do Brasil e iniciar um programa para criar diretrizes.	NA
Automatizar os cartórios para cadastro automático no ConectSus	A ideia é que no momento do registro de um recém-nascido o cartório possa de forma automática criar um cadastro da criança no ConectSus ou gerar um código de acesso que permita o controle da vacinação da criança pelo sistema desde o nascimento.	Cartórios e aplicativo	O tempo todo (deve se tornar rotina diária)	Governo federal e governos estaduais	Criar uma função para interligar os sistemas dos cartórios com o ConectSus.	NA
Combater a desinformação (antivaxx)	Esses grupos radicais estão crescendo rapidamente e a internet tem facilitado a divulgação das informações falsas. Devem ser combatidos antes que a questão se torne ainda mais grave.	Em todo o território nacional	O tempo todo.	Governo federal e governos estaduais	A desinformação se combate com informação confiável, o que só é possível com investimento em pesquisa para geração de conhecimento.	NA
Investimento em pesquisa	Não é possível avançar como sociedade e como país se os investimentos em pesquisas forem suprimidos. A pesquisa gera conhecimento e informações relevantes que determinam como o país deve reagir a determinado problema. O Brasil tem um índice de investimento muito aquém do necessário para geração de conhecimentos relevantes para a sociedade.	Em todo o território nacional (universidades, hospitais escolas, centros de pesquisa etc.)	Deve fazer parte do orçamento anual	Governo federal e governos estaduais	Os governos estaduais e o federal devem aumentar o orçamento para investimento em pesquisa. Considerando os números atuais, é praticamente impossível um avanço significativo nos próximos anos^16^.	NA

NA: Não aplicável

Desconhecimento sobre o HPV e sobre a vacina, hesitação do público, baixo engajamento dos profissionais de saúde, dificuldade de acesso, alcance da promoção e políticas deficientes são as principais causas da baixa adesão à campanha de vacinação no Brasil mostradas no diagrama de Ishikawa. Tais causas foram quantificadas utilizando o gráfico de Pareto ( Figura 2 ), que mostra de forma numérica quais causas devem ser priorizadas em um plano de ação. Para determinar o grau de impacto, foram utilizadas referências de estudos^10 , 11 , 17^que trazem estimativas para cada ponto levantado no eixo X do gráfico de Pareto elaborado. Com as causas classificadas e seu impacto quantificado, foi possível elaborar os resultados da terceira ferramenta de qualidade, utilizada para sugerir propostas de aumento da CV. No gráfico de Pareto, as barras a partir da esquerda mostram os problemas que mais impactam a adesão à campanha de vacinação no Brasil. A linha vermelha mostra o percentual acumulado do impacto na campanha de vacinação. Dessa forma, é possível observar que as causas 1 e 2 têm um impacto acumulado de 55%. Infere-se que o foco aplicado às atenções nas duas primeiras causas poderia resolver os principais problemas que impactam em 55% o aumento da adesão.

O Quadro 2 mostra a proposta de plano de ação utilizando a ferramenta 5W2H, que identifica o que deve ser feito, por que, onde, quando, por quem, como e quanto custa o que deve ser feito. A ferramenta também possibilita transformar cada uma das atividades em métricas para serem controladas e mensuradas. No contexto deste trabalho, os custos das atividades não foram objeto dos resultados, uma vez que são necessárias diversas e complexas informações de custos de saúde pública para calculá-los.

## DISCUSSÃO

Para Austrália, México e Peru, observou-se a boa aplicação e a integração dos quatro “P” conceituais definidos por Kotler como fundamentos das estratégias de marketing social^7 , 8^. O público foi corretamente identificado, o produto gratuito à população facilitou o acesso, o ambiente de aplicação e informação à saúde (escolas) e as táticas de promoção refletiram a necessidade de comunicação objetiva e de fácil aceitação pelo público-alvo de cada país^14 , 18 , 20^. Além disso, observa-se a customização dos materiais de campanha para incluir também uma linguagem acessível aos imigrantes que estão estabelecidos em seus países, como o caso da Austrália, sem fazer acepção de pessoas pelos seus credos, gostos e culturas.

Quanto às ferramentas de qualidade com impacto social, espera-se que o mapeamento das principais causas possibilite a criação de planos para cada uma delas com o objetivo de resolver o problema da baixa adesão. Cada causa tem um impacto significativo na adesão do público-alvo à campanha de vacinação, de forma que prever esse impacto e tomar medidas assertivas contribui para o aumento do engajamento da população e criação da conscientização coletiva, aumentando o CV. O gráfico de Pareto mostra também o percentual de impacto isolado de cada causa. A dificuldade de acesso é mensurada em 34% de acordo com o estudo publicado em 2014 que mostra o impacto do marketing social nas campanhas de vacinação^18^, ou seja, a aplicação do marketing social pode aumentar em 34% a adesão à vacinação. Esse dado é corroborado pelo aumento de 35% de cobertura atingido pelo Uruguai quando mudou a estratégia de vacinação para as escolas.

O alcance da promoção é mensurado no trabalho realizado pelo governo da Austrália, que conseguiu atingir um aumento de até 21%^12^. O desconhecimento sobre a vacina é mensurado em aproximadamente 16% em um estudo realizado no México^19^. Daley et al.^20^afirmam que a recomendação e prescrição pelos profissionais de saúde pode aumentar a adesão em 15%.

De acordo com os dados do estudo de Barretos, a hesitação em vacinar atinge cerca de 8% do público-alvo, deixando o restante do impacto em políticas deficientes em 6%^10^.

## CONCLUSÃO

A realização de etapas consecutivas de revisão bibliográfica e documental, seguida da determinação da real CV (%) no Brasil e da comparação do uso das estratégias de marketing social nas campanhas realizadas por países com alta CV, foi essencial para a aplicação de ferramentas de qualidade que classificaram e quantificaram as causas da baixa CV no Brasil. Por fim, a proposta do plano de ação pormenorizada na ferramenta 5W2H pode ser utilizada na construção de um potencial documento social com propostas baseadas nas boas práticas globais que podem direcionar o MS do Brasil a seguir estratégias mais eficazes para atingir a meta de CV recomendada pela OMS.
